# Diverse metabolic response of cancer cells treated with a ^213^Bi-anti-EGFR-immunoconjugate

**DOI:** 10.1038/s41598-021-84421-4

**Published:** 2021-03-18

**Authors:** Benedikt Feuerecker, Philipp Biechl, Christof Seidl, Frank Bruchertseifer, Alfred Morgenstern, Markus Schwaiger, Wolfgang Eisenreich

**Affiliations:** 1 Department of Nuclear Medicine, School of Medicine, Technische Universität München, Ismaninger Straße 22, 81675 Munich, Germany; 2 Deutsches Konsortium für translationale Krebsforschung (DKTK), Heidelberg, partnersite München and German Cancer Research Center (DKFZ), Heidelberg, Germany; 3 Department of Radiology, School of Medicine, Technische Universität München, Munich, Germany; 4 Department of Chemistry, Bavarian NMR Center-Structural Membrane Biochemistry, Technische Universität München, Garching, Germany; 5 European Commission, Joint Research Centre, Directorate for Nuclear Safety and Security, Karlsruhe, Germany

**Keywords:** Preclinical research, Translational research, Cancer imaging, Cancer metabolism, Cancer therapy

## Abstract

Evaluation of treatment response is among the major challenges in modern oncology. We herein used a monoclonal antibody targeting the EGF receptor (EGFR) labelled with the alpha emitter ^213^Bi (^213^Bi-anti-EGFR-MAb). EJ28Luc (bladder) and LN18 (glioma) cancer cells, both overexpressing EGFR, were incubated for 3 h with the radioimmunoconjugate. To assess the responses in the core carbon metabolism upon this treatment, these cancer cell lines were subsequently cultivated for 18 h in the presence of [U-^13^C_6_]glucose. ^13^C-enrichment and isotopologue profiles of key amino acids were monitored by gas chromatography–mass spectrometry (GC/MS), in order to monitor the impacts of the radionuclide-treatment upon glucose metabolism. In comparison to untreated controls, treatment of EJ28Luc cells with ^213^Bi-anti-EGFR-MAb resulted in a significantly decreased incorporation of ^13^C from [U-^13^C_6_]glucose into alanine, aspartate, glutamate, glycine, proline and serine. In sharp contrast, the same amino acids did not display less ^13^C-enrichments during treatment of the LN18 cells. The data indicate early treatment response of the bladder cancer cells, but not of the glioma cells though cell lines were killed following ^213^Bi-anti-EGFR-MAb treatment. The pilot study shows that the ^13^C-labelling approach is a valid tool to assess the responsiveness of cancer cells upon radionuclide-treatment in considerable metabolic detail.

## Introduction

Evaluation of response to treatment of cancerous diseases is among the major challenges in modern oncology. With [^18^F]FDG-PET, a clinically well-established imaging method is available, which helps to understand and to decipher therapeutic efficacy of anti-tumor therapies in vivo. This method relies on the fact that tumor cells show a high glycolytic turnover combined with the production of vast amounts of lactate, as discovered by Otto Warburg already in the last century^1^. However, as soon as the radioactive glucose analogue [^18^F]FDG gets trapped inside a cancer cell following internalization and enzymatic conversion by hexokinase II, no further metabolic steps can be investigated^2^. In contrast to the ^18^F-method, a labeling approach based on ^13^C-glucose could also glean downstream metabolic processes as monitored by gas chromatography–mass spectrometry (GC–MS) analysis of amino acids derived from glycolytic and citric acid (TCA) cycle intermediates^3,4^. Following a similar approach, metabolic heterogeneity in lung cancers was identified showing higher lactate metabolism compared to benign cells^5–7^. Beyond, pyruvate carboxylation was higher at the site of lung metastases compared to the primary tumor site in breast cancer cells^8^. These examples underline the interplay between different metabolic pathways and metastatic/oncological behavior. The approach using ^13^C-labeled glucose is therefore also a promising concept for the evaluation of targeted therapy, because early treatment responses following incubation of cancer cells with alpha-emitter immunoconjugates still need to be clarified.

In this study, we used an antibody targeting the epidermal growth factor receptor (EGRF) labelled with the alpha emitter ^213^Bi (^213^Bi-anti-EGFR-MAb), to explore treatment associated responses in two different cancer cell lines expressing EGRF. EGFR has been reported to be upregulated on the surface of tumour cells of glioblastoma, lung cancer, head and neck cancer, and bladder cancer. EGFR promotes tumour cell division and tumour growth and, due to its overexpression, it serves as a promising target for targeted therapy^9–12^.

In applying targeted alpha therapy (TAT) using the alpha-emitter ^213^Bi coupled to the anti-EGFR antibody matuzumab, we take advantage of the destructive potential of alpha-emitters as characterized by the high linear energy transfer of alpha-particles^13, 14^. As alpha emitters have only a short range in tissue, it is necessary to warrant a close delivery of the alpha emitter to the target cells. In our study, this is realized using an anti-EGFR-antibody. ^213^Bi-anti-EGFR-MAb selectively eradicates cancer cells that show EGFR overexpression^15^.

We previously showed that hyperpolarized ^13^C enriched pyruvic acid can be used to monitor treatment related changes of ^213^Bi-anti-EGFR-MAb^15^. However, the metabolic turnover of pyruvic acid to lactate is only one of many metabolic steps that occur in a cell, and experimental settings using hyperpolarized ^13^C enriched biomolecules cannot fully resolve metabolic pathways and fluxes in a cell or organ under study. Potential further complications of this approach arise due to unpredictable kinetic rates of metabolic enzymes in the complex environment of human cells and the relatively short half-life of hyperpolarized substrates.

In this study, we have monitored early metabolic response to treatment with ^213^Bi-anti-EGFR-MAb in two different cancer cell lines in vitro via GC–MS-based analysis of ^13^C-glucose incorporation into amino acids. We hypothesize that cells treated with an alpha emitter undergo massive changes of metabolism prior to apoptosis. To date, data on effects of alpha-irradiation upon metabolism of cancer cell is still sparse. Our aim was therefore to use the ^13^C-based metabolic pathway analysis monitored by GC–MS of amino acids to comprehensively determine the impact of an alpha emitter treatment upon the core metabolic pathways in cancer cells. To the best of our knowledge, this is the first study having used the ^13^C-technology to address this question.

## Materials and methods

### Cell lines

The human urothelial carcinoma cell line EJ28Luc, isolated from a primary bladder carcinoma, was grown in RPMI medium supplemented with 10% fetal calf serum and 1% non-essential amino acids (Biochrom, Berlin, Germany) in a humified atmosphere containing 5% CO_2_ at 37 °C. Transfection of cells was previously carried out with plasmid pcDNA3.1 containing the coding sequence of firefly (*Photinus pyralis*) luciferase under the control of the cytomegalovirus promoter^12^. The human glioma cell line LN18 was cultured in RPMI medium supplemented with 10% fetal calf serum. Cells were harvested after rinsing the monolayer with an EDTA/PBS solution (1 mM EDTA in PBS; Biochrom) for 10 min at 37 °C, respectively.

EJ28Luc cells were a gift from Birgit Pfost. LN18 cells were gifted from Jürgen Schlegels’ lab. All methods were carried out in accordance with relevant guidelines and regulations.

Both cell lines were chosen based on the high EGFR expression as deduced from binding of ^213^Bi-anti-EGFR-MAb > 60%, allowing for a targeted treatment. Binding of ^213^Bi-anti-EGFR to both cell lines was shown previously^15^.

### *Coupling of *^*213*^*Bi to an anti-EGFR-MAb*

The anti-EGFR-MAb (matuzumab; Merck, Darmstadt, Germany) was conjugated with the ^213^Bi chelating agent SCN-CHX-A”-diethylenetriaminepentaacetic acid (DTPA) (Macrocyclics, Plano, TX, USA) prior to radiolabelling as previously described^16^. The α-emitter ^213^Bi was eluted from an ^225^Ac/^213^Bi generator system provided by the Directorate for Nuclear Safety and Security (JRC, EC, Karlsruhe, Germany)^12,17^. CHX-A”-DTPA–chelated anti-EGFR-MAb (100 µg) was incubated with the ^213^Bi eluate (37–148 MBq) in 0.4 M ammonium acetate buffer at pH 5.3 for 7 min at room temperature. Unbound ^213^Bi was separated via size-exclusion chromatography using PD-10 columns (GE Healthcare, Munich, Germany). Purity of ^213^Bi-anti-EGFR conjugates was controlled as described earlier^18^. Survival was assessed by microscopical analysis as described previously (see also^15^).

### *Treatment of cells with *^*213*^*Bi-anti-EGFR-MAb and incubation with [U-*^*13*^*C*_*6*_*]glucose*

Cells were seeded in 175 cm^2^ culture flasks (approximately 5 × 10^6^ cells per flask) and allowed to adhere overnight. The next day, cells (approximately 1 × 10^7^ cells per flask) were incubated with lethal activity concentrations of 1.48 MBq/ml of ^213^Bi-anti-EGFR-MAb in a total volume of 10 ml culture medium for 3 h at 37 °C and 5% CO_2_. Controls were treated accordingly, however, an equal volume of PBS was added instead of ^213^Bi-anti-EGFR-MAb solution. Subsequently, culture medium was aspirated and the cells were washed once with 20 ml of PBS. Following addition of 30 ml per flask of [U-^13^C_6_]glucose containing culture medium (glucose-free DMEM [Biochrom, Berlin, Germany] with added unlabelled d-glucose [5 mM], [U-^13^C_6_]d-glucose [5 mM] [99.9% ^13^C-content, Sigma-Aldrich, Taufkirchen, Germany], glutamine [0.1 mM] and 10% FCS), the cells were incubated for another 18 h in the incubator (37 °C, 5% CO_2_). Finally, cells were harvested, washed in PBS three times and the cellular pellets (after removal of the supernatants) were frozen at − 80 °C until further use. Analysis of the cells was done after these 18 h of incubation with [U-^13^C_6_]glucose.

### *Analysis of the *^*13*^*C-content of selected amino acids *via* GC–MS*

To analyse treatment associated metabolic alterations in LN18 and EJ28Luc cells upon exposure to ^213^Bi-anti-EGFR-MAb, mock-treated (controls) and treated cells frozen at − 80 °C were first freeze-dried for 24 h (Martin Christ Gefriertrocknungsanlagen GmbH, Osterode, Germany). For preparation of the amino acids, 1 mL of 6 M HCl was added to 10 mg of freeze-dried cells (approximately 1 × 10^7^) and, after re-suspension, the reaction mixture was incubated for 24 h at 105 °C in a sealed tube. On the following day, the cell hydrolysate was dried at 70 °C in a heat block applying a constant stream of N_2_ gas. To dissolve the residue, 200 μL of 50% acetic acid were added and the sample was sonicated for 1 min. Then, amino acids were purified using a self-made column composed of a 1 mL pipette tip filled with glass wool and the cation-exchange resin Dowex 50 W-X8 (hydrogen form, 200–400 mesh, Alfa Aesar: L13922, Thermo Fisher Scientific, Waltham, Massachusetts, USA). Before applying the sample, the column was washed once with 1 mL 70% methanol and twice with 1 mL H_2_O. After addition of the sample, the column was washed twice with 1 mL H_2_O. The elution of the amino acids was achieved with 1 mL of 4 M NH_3_ solution. A 200 μL-fraction of the 1 mL eluate was transferred to a glass vial and dried at 70 °C using N_2_ as described above. The remaining 800 µL of the eluate were kept at − 20 °C for replicate analyses. Amino acids were converted into volatile silyl-derivatives and subjected to GC/MS-analysis, as described earlier^19,20^. More specifically, 50 μL of water-free acetonitrile (ACN) and 50 μL of *N*-methyl-*N-tert*-butyldimethylsilyltrifluoroacetamide (MTBSTFA) containing 1% TBDMS chloride were added and the samples were incubated for 30 min at 70 °C in a sealed GC–MS vial. Finally, samples were transferred to GS–MS microvials and detection of amino acids was done using a GC–MS instrument (GC 2010, GCMS-QP 2010, auto injector AOC-20i, Shimadzu, Munich, Germany).

### Data analysis

GC/MS results were evaluated with the associated software GCMSsolution from Shimadzu. Further analysis employed statistical analysis using GraphPad Prism Version 5 (GraphPad Software, Inc., USA). T-Tests were performed followed by Welch’s test. Statistical significance was assumed if p was < 0.05.

## Results

### Radiochemical yield, specific activity and purity

After a 7-min incubation of the ^213^Bi eluate with anti-EGFR-MAb, the labeling yield varied between 95 and 97% of ^213^Bi bound to anti-EGFR-MAb, the respective specific activities were 0.35–1.4 MBq of ^213^Bi per mg of antibody. After removal of unbound ^213^Bi via size-exclusion chromatography, a purity of ^213^Bi-anti-EGFR-MAb of greater than or equal to 99% was achieved. The in vitro stability of ^213^Bi-anti-EGFR-MAb exceeded four half-lives of ^213^Bi (3 h), which is in accordance with previous data (see also^12^).

### *Effect of *^*213*^*Bi-anti-EGFR-MAb treatment on survival of tumor cell lines*

The activity concentration of ^213^Bi-anti-EGFR-MAb applied for incubation of both LN18 glioma and EJ28-luc bladder cancer cells (1.48 MBq/mL) proved to be lethal for approximately 99% of the cells. However, the 3-h treatment with the alpha-emitter immunoconjugate did not eradicate the cells immediately. Cells started to disintegrate not before 72–96 h after incubation with ^213^Bi-anti-EGFR-MAb and cell death was completed approximately after 120 h. At the time of analysis of the ^13^C-content of selected amino acids, i.e. 48 h after treatment with ^213^Bi-anti-EGFR-MAb, cells were still alive, however showed morphological changes compared to mock-treated controls. The most striking difference as observed 48 h after incubation was a massive swelling of the ^213^Bi-anti-EGFR-MAb treated cells compared to mock-treated controls (data not shown). Likewise, cells that survived ^213^Bi-anti-EGFR-MAb treatment (approximately 1%) initially underwent a stationary phase of intensive DNA-repair without cell proliferation, lasting approximately 48–72 h.

### ^*13*^*C-Enrichment and isotopologue composition in selected amino acids*

**Figure 1:**
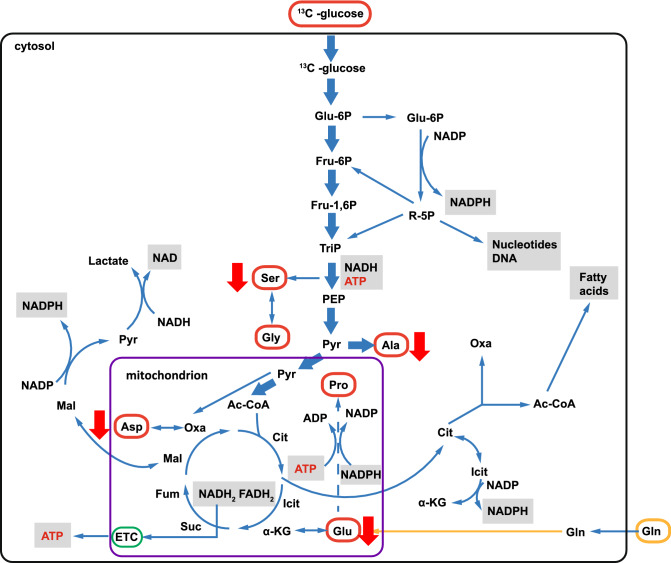
Cellular metabolism of glucose. The figure shows relevant steps in metabolization of glucose following uptake from the extracellular milieu. Red circles indicate the analyzed amino acids, i.e. alanine (Ala), aspartate (Asp), glutamate (Glu), proline (Pro), serine (Ser), and glycine (Gly), with regard to enrichment of ^13^C in mock-treated controls and cells incubated with ^213^Bi-anti-EGFR-MAb. Results of ^13^C-enrichment in EJ28Luc bladder cancer cells. Red arrows indicate significantly decreased enrichment of ^13^C in ^213^Bi-anti-EGFR-MAb treated cells compared to controls. ETC electron transport chain; (adapted from^4^).

As examples for EGFR-overexpressing cancer cells, we have selected the bladder cancer cell line EJ28-Luc and the glioma cell line LN18^15^. These cell lines were incubated for 3 h with ^213^Bi-anti-EGFR-Mab and subsequently for 18 h with [U-^13^C_6_]glucose. After that, the cells were hydrolysed under acidic conditions. The hydrolysate containing alanine, aspartate, glutamate, glycine, proline and serine was silylated and analysed by GC–MS analysis. By careful evaluation of the mass patterns, the relative abundances of amino acids carrying a given number of ^13^C-atoms were determined resulting in the isotopologue patterns and overall ^13^C-contents reported below. Notably, the amino acids under study derive from glycolytic intermediates, such as 3-phosphoglycerate (serine and glycine), pyruvate (alanine), oxaloacetate (aspartate), or α-ketoglutarate (glutamate and proline) following well established pathways in human metabolism (see also Fig. 1). Capitalizing on this fact, impacts of the treatment with ^213^Bi-anti-EGFR-MAb upon early glucose metabolism (i.e. glycolysis) and downstream carbon fluxes (i.e. citrate cycle) become transparent by this approach.

### *Overall *^*13*^*C-contents of amino acids*

**Figure 2:**
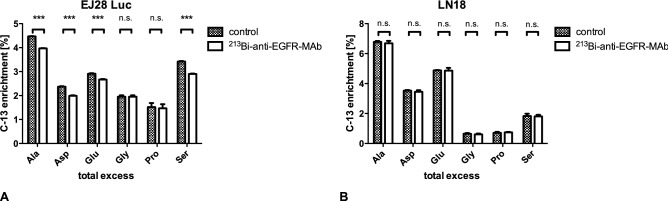
Overall enrichment of ^13^C in analyzed amino acids of untreated control cells and ^213^Bi-anti-EGFR-MAb treated cells. Results are shown for EJ28-Luc bladder cancer cells (**A**) and LN18 glioma cells (**B**). ^213^Bi-anti-EGFR-MAb treatment of EJ28-Luc cells resulted in a significant decrease of ^13^C-enrichment in alanine, aspartate, glutamate, and serine (***p < 0.0001 compared to controls). Moreover, we observed a slight (nonsignificant = n.s.) decrease in proline. Enrichment of ^13^C varied from 1.6% in proline to 4.5% in alanine (**A**). In contrast, no significant changes were observed for LN18 cells.

In EJ28Luc bladder cancer cells, incubation with ^213^Bi-anti-EGFR-MAb resulted in a significant decrease of overall ^13^C-incorporation into alanine (3.9% vs 4.5% in controls), into serine (2.9% vs 3.4% in controls), into glutamate (2.7% vs 2.9% in controls), and into aspartate (1.9% vs 2.4% in controls) (Fig. 1, red arrows; Fig. 2A). In contrast, LN18 glioma cells showed no significant decrease of ^13^C-incorporation into these amino acids (Fig. 2B).

### Isotopologue profiles of amino acids from EJ28Luc cells

**Figure 3:**
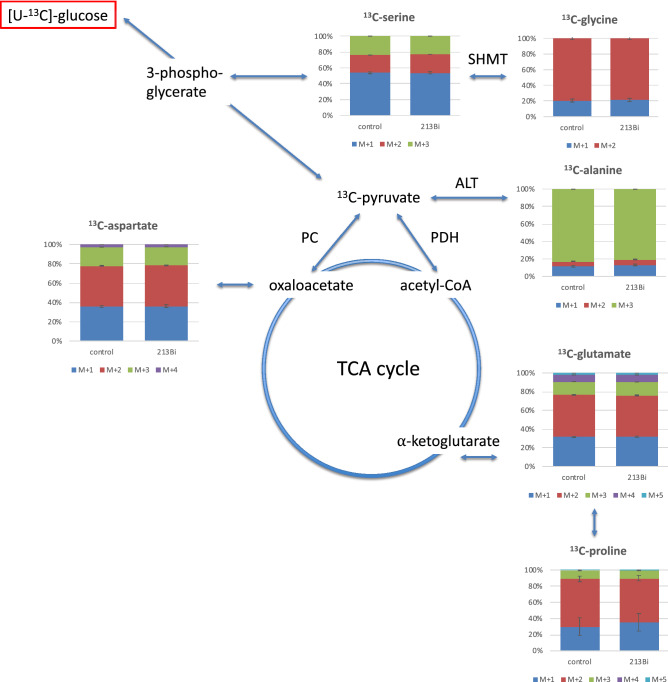
Isotopologue profiling with regard to ^13^C in alanine, aspartate, glutamate, glycine, proline, and serine following incubation of EJ28Luc cells with ^213^Bi-anti-EGFR-MAb compared to untreated controls. Just as in Fig. 2 showing overall enrichment of ^13^C, a significantly lower enrichment of ^13^C in the various C-atoms following ^213^Bi-anti-EGFR-MAb treatment was only observed for alanine, aspartate, glutamate, and serine but not for glycine and proline. *SHMT* serine hydroxymethyl transferase, *ALT* alanine aminotransferase, *PC* pyruvate carboxylase, *PDH* pyruvate dehydrogenase.

The most abundant isotopologue of alanine was the [U-^13^C_3_]-species containing three (out of three) ^13^C-atoms (also denoted M + 3, since this isotopologue is characterized by an increase of three mass units due to three ^13^C-atoms in comparison to the unlabelled species carrying three ^12^C-atoms (Fig. 3 and Table 1). Glycine, aspartate, glutamate and proline showed the M + 2 isotopologues as the most abundant ones, whereas M + 1 was the dominant one in serine (Fig. 3). The relative amounts of these isotopologues were significantly reduced in ^213^Bi-anti-EGFR-MAb treated EJ28Luc cells compared to untreated controls in case of alanine (3.6% vs 4.1%), aspartate (1.8% vs 2.12%), glutamate (2.9% vs 3.2%), and serine (2.8% vs 3.2%). However, the differences shown for glycine and proline were not significant (Fig. 3 and Table 1).

**Table 1:** Percentage of ^13^C-enrichment and enrichment profiles in selected amino acids in controls and EJ28Luc and LN18 cell lines after treatment with ^213^Bi-anti-EGFR-MAb.

	EJ28Luc		LN18	
	Control	^213^Bi-anti-EGFR-MAb	Control	^213^Bi-anti-EGFR-MAb
**Alanine**				
M + 1	0.56% ± 0.04%	0.59% ± 0.04%	0.66% ± 0.02%	0.63% ± 0.03%
M + 2	0.30% ± 0.01%	0.28% ± 0.01%	0.44% ± 0.00%	0.40% ± 0.02%
M + 3	4.09% ± 0.02%	3.58% ± 0.02%***	6.27% ± 0.05%	6.21% ± 0.14%
**Aspartate**				
M + 1	1.79% ± 0.06%	1.53% ± 0.06%**	1.97% ± 0.06%	1.81% ± 0.10%
M + 2	2.12% ± 0.02%	1.78% ± 0.02%***	2.78% ± 0.08%	2.75% ± 0.08%
M + 3	0.98% ± 0.00%	0.82% ± 0.00%***	1.84% ± 0.03%	1.83% ± 0.06%
M + 4	0.13% ± 0.01%	0.10% ± 0.01%*	0.27% ± 0.01%	0.25% ± 0.01%
**Glutamate**				
M + 1	2.27% ± 0.04%	2.08% ± 0.04%**	2.54% ± 0.00%	2.49% ± 0.07%
M + 2	3.22% ± 0.03%	2.88% ± 0.03%***	5.57% ± 0.03%	5.56% ± 0.18%
M + 3	1.01% ± 0.01%	0.95% ± 0.01%**	1.70% ± 0.05%	1.73% ± 0.09%
M + 4	0.52% ± 0.00%	0.48% ± 0.00%***	0.95% ± 0.01%	0.94% ± 0.05%
M + 5	0.15% ± 0.00%	0.14% ± 0.00%***	0.36% ± 0.01%	0.35% ± 0.02%
**Glycine**				
M + 1	0.45% ± 0.04%	0.47% ± 0.04%	0.23% ± 0.03%	0.22% ± 0.03%
M + 2	1.72% ± 0.04%	1.72% ± 0.04%	0.54% ± 0.04%	0.50% ± 0.04%
**Proline**				
M + 1	1.26% ± 0.45%	1.49% ± 0.45%	0.42% ± 0.03%	0.41% ± 0.03%
M + 2	2.47% ± 0.14%	2.30% ± 0.14%	1.24% ± 0.15%	1.33% ± 0.06%
M + 3	0.46% ± 0.03%	0.42% ± 0.03%	0.21% ± 0.02%	0.21% ± 0.00%
M + 4	0.00% ± 0.00%	0.00% ± 0.00%	0.00% ± 0.00%	0.00% ± 0.00%
M + 5	0.00% ± 0.00%	0.00% ± 0.00%	0.00% ± 0.00%	0.00% ± 0.00%
**Serine**				
M + 1	3.24% ± 0.07%	2.76% ± 0.07%**	1.52% ± 0.24%	1.94% ± 0.16%
M + 2	1.32% ± 0.00%	1.19% ± 0.00%***	0.67% ± 0.07%	0.68% ± 0.04%
M + 3	1.45% ± 0.00%	1.18% ± 0.00%***	0.87% ± 0.04%	0.71% ± 0.05%*

*p < 0.05, **p < 0.01, ***p < 0.0001 compared to controls.

### Isotopologue profiles of amino acids from LN18 cells

**Figure 4:**
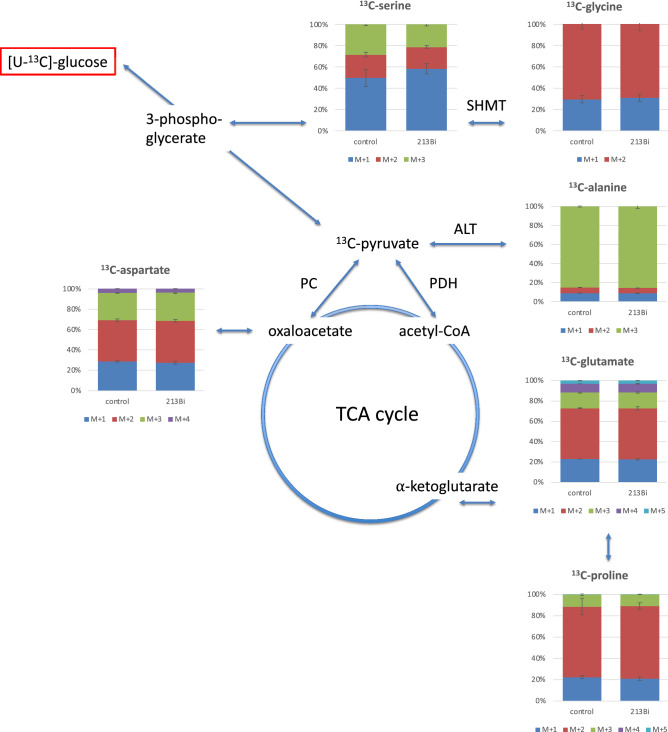
Isotopologue profiling with regard to ^13^C in alanine, aspartate, glutamate, glycine, proline, and serine following incubation of LN18 cells with ^213^Bi-anti-EGFR-MAb compared to untreated controls. As expected from the results shown in Fig. 2, isotopologue profiling did not reveal significant differences between ^213^Bi-anti-EGFR-MAb treated and untreated LN18 cells, except for the serine isotopologue containing three ^13^C-atoms (M + 3). However, the frequency of the different ^13^C isotopologues was identical for all amino acids in both cell lines analyzed (EJ28Luc and LN18). For example, in proline consisting of five C-atoms, the most frequent isotopologues contained two ^13^C-atoms and isotopologues with four or five ^13^C atoms could not be detected in both cell lines. *SHMT* serine hydroxymethyl transferase, *ALT* alanine aminotransferase, *PC* pyruvate carboxylase, *PDH* pyruvate dehydrogenase.

In LN18 glioma cells, isotopologue profiling could not reveal significant differences in ^13^C-enrichments (Fig. 2B) but also not in the isotopologue compositions of the selected amino acids following ^213^Bi-anti-EGFR-MAb treatment compared to untreated controls (Fig. 4 and Table 1). More specifically, the relative fractions of these isotopologues were not significantly reduced in case of alanine with three ^13^C labelled atoms (6.3% vs 6.2%), two (out of four) labelled ^13^C atoms in aspartate (2.8% vs 2.8%) and two (out of five) labelled ^13^C atoms in glutamate (5.6% vs 5.6%). Accordingly, the differences shown for glycine and proline were not significant. Only the serine isotopologue containing three ^13^C-atoms showed small, but significant differences (1.9% vs 1.5%; Fig. 4).

## Discussion

Treatment of cells showing high EGFR expression was chosen so that the ^213^Bi-anti-EGFR-MAb could bind to the surface of tumor cells, therefore taking advantage of the high linear energy transfer of the alpha emitter ^213^Bi^13^^,^^14^. We have shown previously that this targeted approach results in effective cell death of both LN18 and EJ28Luc cells^15^. Other studies revealed that external irradiation can induce enhanced EGFR and MMP-2 secretion in LN18 cells after irradiation with doses ranging from 0.5 to 15 Gy using 6 MV X(-)rays^21^, thus underlining the principle effect of irradiation in glioma cells. Alpha-particle irradiation with Americium-241, emitting α-particles with an energy of 5.49 meV (LET = 85 keV/μm) at 1.3 Gy resulted in a decrease (albeit not statistically relevant) of relative invasion in an in vitro assay^22^. In patients with bladder carcinoma overexpressing EGFR, treatment with an ^213^Bi-anti-EGFR-MAb-conjugate resulted in satisfactory therapeutic efficacy, showing that in three of 12 patients no signs of carcinoma in situ were present at 3, 30 and 44 months after treatment^23^. Yet, the effects of irradiation using the alpha emitter ^213^Bi on cell metabolism at a molecular level has not been studied in detail.

Among the hallmarks of cancer is that cancer cells show a high glycolytic rate and lactate production, an anomalous characteristic of cell energy metabolism first described by Otto Warburg^24^. Cancer cells limit their energy production largely to glycolysis even though oxygen is present by reprogramming their glucose metabolism^25^. In glioma, a relevant proportion of cell metabolism is supplied through glutamine metabolism, resulting in a so called “glutamine addiction”^26^.

In our experimental setting, EJ28Luc bladder cancer and LN18 glioma cell lines were cultivated in a medium containing 5 mM of [U-^13^C_6_]glucose and 5 mM of unlabelled glucose as well as 0.1 mM glutamine. Given the composition of this cell medium, glycolytic flux might be preferred by the cells due to the low glutamine concentrations. On the other hand, the chosen growth conditions allowed for direct insights into glycolytic flux as a potential early metabolic response upon treatment with ^213^Bi-anti-EGFR-MAb. Removing glutamine entirely from the medium is challenging as cells deteriorate quickly. In human fibroblasts deficiency of glutamine but not glucose results in apoptosis as shown previously^27^.

The experimental approach which was used in this study exploits the GC/MS-based ^13^C-analysis of amino acids for the study of metabolic pathway and fluxes^4^. Isotopologue patterns and the overall ^13^C-content in amino acids from the untreated cancer cells under study indeed reflected the ^13^C-fluxes from the offered ^13^C-glucose into the respective precursors of these amino acids which also represent central metabolic intermediates involved in glycolysis or the citrate (TCA) cycle (see also Fig. 1).

The labelling data of these untreated cells suggest that glucose uptake and usage via glycolysis and the citrate cycle is more effective in LN18 cells compared to EJ28Luc cells, as gleaned from the higher ^13^C-content in alanine, aspartate, and glutamate from the ^13^C-labelled LN18 cells in comparison to the EJ28Luc cells (see Fig. 2). On the other hand, the ^13^C-content in serine and glycine was lower compared to EJ28Luc cells, possibly indicating a higher use of external (unlabelled) serine by the LN18 cells. This could save NAD+ as serine production from glucose involves two oxidation reactions, which consume NAD+ and produce NADH^28^. It was shown in A549 cells, in MDA-MB-468 cells, and in MDA-MB-231 cells that these cancer cells need sources of exogenous serine for maximal proliferation^28^.

With regard to proline, the enrichment was lower in LN18 cells compared to EJ28Luc cells. This could potentially indicate a facilitated metabolism form arginine via ornithine. We hypothesize that this might be necessary for nitrogen mobilization or to provide energy from the TCA cycle. Yet, the direct connection to therapy response is missing.

The ^13^C-pattern of alanine is of special interest since its direct precursor is pyruvate, the product of glycolysis. Thus, alanine is synthesized from pyruvate by alanine aminotransferase (ALT), hereby inheriting the isotopologue distribution from pyruvate. As pyruvate is mainly synthesized via glycolysis originating from [U-^13^C_6_]glucose, the predominant [U-^13^C_3_]-isotopologue (M + 3) can be easily understood. Other isotopologues (M + 2 and M + 1) are mainly due to anabolic fluxes from oxaloacetate via gluconeogenesis or from malate via catalytic action of the malic acid enzyme (see Fig. 1). More specifically, phosphoenolpyruvate carboxykinase (PEPCK) provides phosphoenolypyruvate, which can be converted into pyruvate. Consequently, alanine inherits the M + 2 and M + 1 labeling pattern from oxaloacetate. These isotopologues were indeed observed in aspartate (the amination product of oxaloacetate) but also in serine and glycine supporting some metabolic flux via gluconeogenesis although glucose was abundant in the medium. From the observed ratio between M + 3 and M + 2/M + 1 alanine, it can be concluded that glycolysis was the driving force in the malignant cancer cells under study and that fluxes via gluconeogenesis or the malic acid enzyme were negligible (see Table 1).

Quite surprisingly, the impacts of the treatment with ^213^Bi-anti-EGFR-MAb upon the ^13^C-labelling patterns described above were highly different for the cell lines under study. Only EJ28Luc cells showed a treatment related decrease of glycolytic flux after incubation with ^213^Bi-anti-EGFRA-MAb with a statistically significant lower total excess in alanine, aspartate and glutamate enrichment (Fig. 2). Based on the higher glycolytic flux in the LN18 cells and no measurable effects upon the ^13^C-profiles due to treatment with ^213^Bi-anti-EGFR-MAb, LN18 cells might in part show a treatment resistance on a metabolic level at least under the experimental conditions used in this study. In line with this hypothesis is that high glucose uptake above the biomass requirements results in an excess production of redox cofactors linked to stress resistance^29^. In a high-grade human ovarian adenocarcinoma model, it was demonstrated that the cells accumulate micro RNA of the family of miR-200, have low concentrations of p38a, and an associated oxidative stress signature that showed better response to paclitaxel chemotherapy, which is known to increase reactive oxygen species^30^. However, given that the tumor cells show apoptosis after incubation with ^213^Bi-anti-EGFR-MAb, it is more likely that disturbance in other processes not related to metabolism lead to lethal cell damage. With the applied methods these processes may not be displayed.

The observation that only the ^13^C-profiles of EJ28Luc cells showed statistically significant changes due to treatment with ^213^Bi-anti-EGFR-MAb might indicate that EJ28Luc cells die faster than LN18 cells. A potential reason for this could be that LN18 cells are not as sensitive towards irradiation with the ^213^Bi-anti-EGFR-MAb immunoconjugate, albeit the fact that these cells eventually deteriorate over time, as shown previously^15^. This fits to the previous observation that [^18^F]FDG uptake did not decrease statistically significant in LN18 cells, however in EJ28Luc cells after irradiation with a ^213^Bi-anti-EGFR-MAb^15^. Interestingly, [^18^F]FDG uptake was slightly higher in LN18 cells compared to EJ28 Luc cells prior to treatment (ca. 38% vs. ca. 30%)^15^. The latter finding might be a result of enhanced glucose (GLUT) transporter capacity. It was shown in human and murine glioblastoma cells that by inhibition of GLUT/SLC2A with indinavir, ritonavir and by inhibition of the Na/glucose antiporter type 2 (SGLT2/SLC5A2) superfamily with phlorizin, glucose consumption and cell proliferation were decreased^31^. In C6 glioma cells, glutamine metabolism appeared complementary to that of glucose with respect to energy production as carbon donor to replenish the tricarboxylic acid cycles^32^. In order to sustain TCA cycle function, oxaloacetate needs to be refilled by anapleurosis^33^, for example by carboxylation of pyruvate or phosphoenol pyruvate. This allows cells to use the TCA cycle as a supply of biosynthetic precursors^33^. In glioma, metabolism from pyruvate to oxaloacetate seems to be decreased as indicated by the ratio of pyruvate carboxylase/PDH activity compared to normal glia and neuronal tissue^34^. In cancer cells, a more relevant anaplerotic pathway appears to be driven by the usage of glutamine, which contributes to proliferation and biosynthesis^35,36^. Generally, in proliferating glioma cells glutamine is mainly utilized for anapleurosis as carbon donor to replenish the tricarboxylic acid cycle as demonstrated by ^13^C nuclear magnetic resonance spectroscopy in C6 glioma cells^32^ and in human SF188 glioblastoma cells^37^. In cells depleted of glutamine, pools of fumarate, malate and 5-oxo-proline were significantly decreased and these cells went into apoptosis^27^.

Interestingly, EJ28Luc and LN18 cells showed highly similar relative abundance of the different ^13^C isotopologues. Given that both cell lines represent totally different cancer types, this is noteworthy, allowing potentially for a similar therapeutic approach.

Using the approach with [U-^13^C_6_]glucose, deeper insights into cellular metabolism might become possible beyond the first step of glycolysis as accessible with [^18^F]FDG-PET imaging, since [^18^F]FDG gets trapped after phosphorylation through hexokinase. Moreover, approximately 30% of cancers cannot be detected by [^18^F]FDG-PET, either because these tumors do not show a glucose uptake that is above the limit of detection or the tumor entity relies on other metabolic pathways to fuel energy demands^38^.

Further steps of the glycolytic pathway are not directly accessible using [^18^F]FDG-PET imaging. A possible approach to gain further insights is the use of ^13^C-enriched glucose or intermediates of glycolysis such as ^13^C-labeled pyruvate. Labelling with ^13^C takes advantage of the fact that naturally ^13^C is low in abundance (ca. 1%)^39^ and therefore can be used to investigate metabolism using magnetic resonance spectroscopy (NMR). At thermal equilibrium, these measurements require relatively long time to obtain a reasonable signal to noise ratio (SNR) due to the inherently low sensitivity of NMR. Dynamic nuclear hyperpolarization showed to improve SNR by > 10,000-fold and allowed for in vitro and in vivo characterization of metabolic processes^40^. Among the most frequently used substances is ^13^C-pyruvate, which allows to describe metabolic alterations of cancer cells through measurement of pyruvate to lactate metabolism in vitro and in vivo^15,41–43^. In principle, this is also possible with hyperpolarized ^13^C-enriched glucose as shown before^44^. However, the short T1 time of 8–10 s of pre-deuterated hyperpolarized ^13^C-enriched glucose makes it very challenging to follow several steps of glycolysis in vitro and in vivo using NMR^45^. Within this short time interval, the substance has to be dissolved and has to be given to the cells or patients. Another approach was recently proposed using chemical shift imaging (CSI) experiments using magnetic resonance imaging (MRI)^46^. By improving the post-processing procedure by higher dimensional analog of singular value decomposition, tensor decomposition, an approximately 30-fold increase of SNR could be achieved in CSI experiments^46^. We plan to establish such a protocol in future in order to be able to take advantage of this approach. To further explore which metabolic intermediates might be potentially interesting for such a “metabolic” approach, we also aimed to understand the metabolic roads of glycolytic intermediates using the ^13^C-approach monitored by GC–MS. This approach starting from [U-^13^C_6_]glucose allows for a precise quantification of cell metabolism without additional hurdles of fast decay of signals as for instance with hyperpolarized ^13^C-glucose. Given that EJ28Luc cells show changes of glycolytic intermediates leading to amino acids under study, this may enable future “focused” studies using also hyperpolarized ^13^C glucose or non-hyperpolarized magnetic resonance spectroscopy using CSI.

Beyond these aspects, the pilot experiments in this study show the effects of the alpha-emitter ^213^Bi upon cell metabolism in considerable detail. This is of relevance, as it was shown previously that metformin and temozolomide enhanced the effectiveness of photon irradiation in LN18 glioblastoma cells^47^. This coincided with G2/M cell cycle arrest and changes in pAMPK levels possibly triggering cell metabolism^47^. As treatment of glioblastoma remains to be challenging, identification of new “metabolic” targets might provide new treatment options. Combination of external or internal radiation with targeted therapies might enhance treatment success. In the invasive bladder carcinoma cell line EJ28, it was demonstrated that, in varying conditions of confluence and hypoxia (0.1% O_2_), hypoxia significantly increased VEGF protein expression, which correlated with expression of the transcription factors hypoxia inducible factor (HIF) HIF1-alpha and HIF-2-alpha having again impacts on the core metabolism of these cells^48^.

By a better understanding of treatment related effects on a metabolic level, new therapeutic targets could be identified. However, the ^13^C-approach described here has limitations. For example, a direct monitoring using intact cells is still not possible. Additionally, the method requires a significant amount of time in order to prepare and analyse treatment-related effects even under in vitro conditions. This might limit its broad application in a pre-clinical and clinical setting. On the other hand, also [^18^F]FDG-PET imaging in glioma and also in bladder cancer cannot be easily applied: human brain metabolism largely relies on glucose metabolism and therefore shows high background uptake of [^18^F]FDG; besides [^18^F]FDG is excreted via the urinary tract and therefore accumulates in the bladder, resulting in the difficulty of identifying the tumour. In the case of glioma, the use of 4-^18^F-(2S, 4R)-fluoroglutamine was proposed for PET imaging of glioma, offering the advantage of low background uptake through imaging of glutamine uptake^49^.

To our knowledge, this is the first study that evaluated the effects of alpha-irradiation using ^213^Bi-anti-EGFR-MAb on cellular metabolism. Targeted treatment with alpha emitters is recently gaining more interest, e.g. for prostate cancer using ^225^Ac-PSMA-617. Nevertheless, studies show that although initial good treatment effects can be achieved, some patients show progressive disease eventually^50–52^. Thus, our results underscore the importance of a better understanding of cellular metabolism with the aim of potentially identifying new targets for combination treatments to enhance treatment success.

## Conclusions

Treatment of EJ28Luc bladder cancer cells, but not of the LN18 glioma cells with ^213^Bi-anti-EGFR-MAb resulted in decreased incorporation of ^13^C-labelled glucose into amino acids derived from glycolytic and TCA cycle intermediates, indicative of an early treatment response. Our pilot experiments show that the ^13^C-labelling approach can be a valid tool to assess the responsivity of cancer cells upon targeted alpha radionuclide treatment at high metabolic resolution.

## Data Availability

Data generated or analyzed during this study are included in this published article.
